# Peer Review of “Insider Threats to the Military Health System: A Systematic Background Check of TRICARE West Providers”

**DOI:** 10.2196/57701

**Published:** 2024-04-09

**Authors:** Anonymous

**Keywords:** TRICARE, health care fraud, Defense Health Agency, fraud, fraudulent, insurance, coverage, beneficiary, beneficiaries, background check, background checks, demographic, security clearance, FDA, Medicaid, Medicare, provider, provider referral, military, False Claims Act, HIPAA breach, OIG-LEIE, inspector general, misconduct, insider threat, information system, zero trust, data management, Food and Drug Administration, Health Insurance Portability and Accountability Act breach, Office of Inspector General's List of Excluded Individuals and Entities

*This is the peer-review report for “Insider Threats to the Military Health System: A Systematic Background Check on TRICARE West Providers.”*

## Round 1 Review

### General Comments

In general, the manuscript [1] is informative and includes a lot of information on health care providers who participate in TRICARE insurance. The study examines those who have received some sort of exclusion, sanction, or other reprimand based on health care fraud or harm. This study is timely and has practical implications for protecting patient care, particularly for those who are in a vulnerable position such as veterans or warfighters. I hope the following comments are taken as constructive criticism and interest in the overall improvement of the study. I appreciate the opportunity to review this study.

Below is a list of important fixes that I recommend considerable time be spent on and some minor fixes. In general, I think the key limitation of the study is that it can better state the significant contribution of the study. I understand the need for such a study, but as it stands, the study can further improve by spending more time on why and how health care providers land on exclusion lists such as the Office of Inspector General’s (OIG) List of Excluded Individuals and Entities (LEIE). Indeed, the study uses several databases that exclude physicians or provide reasons why a physician no longer participates in such programs, but the author can improve their justification for the study on why this is needed.

The second key limitation of the study is the Methods and Results section. In particular, this section needs improvement with clearer detail and justification on why the author had a selection criterion (vs examining all zip codes). In addition, the Results section can improve with better organization of the findings. As it reads, the results are a bit difficult to follow with all the zip codes laid out.

Last, the study could benefit from greater discussion on the implications of the study. At the moment, it pushes for more transparency, but the author could use their data more to discuss the impact of their findings. For example, why would publishing the National Provider Identification (NPI) numbers help patients? What do patients or the author want to gain from that transparency? How can this help future patients or hold physicians more accountable? The discussion loosely taps into the implications, but the study could really tease out this argument more.

Overall, the study was easy to follow and did provide some interesting content to consider. I think the study can better serve the public and has great implications! I would like to see these implications highlighted more so that the reader can really see the contribution the study makes.

### Specific Comments

#### Major Comments

1. Introduction: Provide an explanation of what the OIG LEIE is for the reader. It is important to inform the reader that the OIG LEIE excludes participation in the program for various reasons—not just a quality-of-care issue. For example, the OIG LEIE also can exclude physicians on a financial offense matter. This helps the reader understand the gravity of the situation, particularly when the author discusses the increased risk of mortality and hospitalization of these patients. In short, I would like to see more development and background on how individuals are included in the LEIE to increase awareness for the reader.
  - For more information about LEIE and how physicians are placed on the list, please see the following: Burton B, Sun D, Jesilow P, Pontell HN. Two paths, one destination: a demographic portrait of physicians sanctioned by the federal government. *J Health Hum Services Adm*. 2022;45(3):142-180.
2. Methods: This section needs improvement. First, please provide more justifications and in-text citations to justify the methods used for the study. This will help strengthen the Methods section. As it reads now, there seem to be no prior studies listed that use this method (although that is not the case). Second, why did the author limit the search to the “83 most populous zip codes”? Why not include all zip codes? Does this relate to the number of people participating in TRICARE, or is this because there are simply more people living in those zip codes? Please include a justification here on why there is a population cutoff. Third, on page 8, the author writes that there were 22 states that were included, but the list only included 21 states (from my understanding). In addition, why were some of the zip codes (eg, St. Louis, Rock Island Arsenal) excluded and others included (eg, Amarillo, Lubbock, and El Paso areas only for Texas)?
3. Results: The author generally states that their findings are consistent with past research but do not include a list of articles to which they are referring. Only one article is referenced [2]. Please provide further support for that claim (in other words, please include all other studies to support the claim of consistent findings). In addition, when discussing results like on page 10, the presentation is difficult to follow with all the zip codes listed and separated by a hyphen. Please consider reorganizing this presentation or placing the list of zip codes in a footnote to ease the presentation of results.
4. Discussion: The significance of the study could be further elaborated on. At the moment, it pushes for more transparency, but the author could use their data more to discuss the impact of their findings. For example, why would publishing the NPI numbers help patients? What do patients or the author want to gain from that transparency? How can this help future patients or hold physicians more accountable? The discussion loosely taps into the implications, but the study could really tease out this argument more.

#### Minor Comments

1. Clean up the grammar and punctuation. For example, on page 4, the author states, “Nicholas et al performed a cross-sectional study of 8204 Medicare beneficiaries who received care from excluded providers. It revealed that patients treated by fraudsters experience a 13%-23% increased risk of mortality and 11%-30% higher risk of hospitalization (Nicholas et al, 2019).” Note, that the start of the sentence, “Nicholas et al” needs a period and a year in the citation.
2. I suggest adding a numerical list when discussing the different databases that are available for searching a physician. For example, on page 5, the author lists several different databases starting with the sentence “Multiple public databases exist to search names with respect to each of these issues, including...” Adding in a numbered list can make the information more digestible for the audience. This can also be cleaned up (ie, adding a numeric list) on page 7 when listing the different databases that the physicians were screened in.
3. Page 6, it is stated that 203 names appeared in up to 3 additional types of databases. However, what are these 3 additional types of databases? Is it referring to the earlier-mentioned databases? This is unclear.

## Abbreviations

LEIE: List of Excluded Individuals and Entities
NPI: National Provider Identification
OIG: Office of Inspector General
